# Modeling Saccadic Action Selection: Cortical and Basal Ganglia Signals Coalesce in the Superior Colliculus

**DOI:** 10.3389/fnsys.2019.00003

**Published:** 2019-02-13

**Authors:** Brian C. Coe, Thomas Trappenberg, Douglas P. Munoz

**Affiliations:** ^1^Centre for Neuroscience Studies, Queen's University, Kingston, ON, Canada; ^2^Faculty of Computer Science, Dalhousie University, Halifax, NS, Canada

**Keywords:** eye movements, inhibition, anti-saccade, dynamic neural field model, reaction time, error rate, human behavior

## Abstract

The distributed nature of information processing in the brain creates a complex variety of decision making behavior. Likewise, computational models of saccadic decision making behavior are numerous and diverse. Here we present a generative model of saccadic action selection in the context of competitive decision making in the superior colliculus (SC) in order to investigate how independent neural signals may converge to interact and guide saccade selection, and to test if systematic variations can better replicate the variability in responses that are part of normal human behavior. The model was tasked with performing pro- and anti-saccades in order to replicate specific attributes of healthy human saccade behavior. Participants (ages 18–39) were instructed to either look toward (pro-saccade, well-practiced automated response) or away from (anti-saccade, combination of inhibitory and voluntary responses) a peripheral visual stimulus. They generated express and regular latency saccades in the pro-saccade task. In the anti-saccade task, correct reaction times were longer and participants occasionally looked at the stimulus (direction error) at either express or regular latencies. To gain a better understanding of the underlying neural processes that lead to saccadic action selection and response inhibition, we implemented 8 inputs inspired by systems neuroscience. These inputs reflected known sensory, automated, voluntary, and inhibitory components of cortical and basal ganglia activity that coalesces in the intermediate layers of the SC (SCi). The model produced bimodal reaction time distributions, where express and regular latency saccades had distinct modes, for both correct pro-saccades and direction errors in the anti-saccade task. Importantly, express and regular latency direction errors resulted from interactions of different inputs in the model. Express latency direction errors were due to a lack of pre-emptive fixation and inhibitory activity, which aloud sensory and automated inputs to initiate a stimulus-driven saccade. Regular latency errors occurred when the automated motor signals were stronger than the voluntary motor signals. While previous models have emulated fewer aspects of these behavioral findings, the focus of the simulations here is on the interaction of a wide variety of physiologically-based information integration producing a richer set of natural behavioral variability.

## Introduction

Saccadic action-selection requires a complex network of brain areas that provide different information signals (e.g., sensory, motor, cognitive). In a pro- and anti-saccade task participants are asked to either look toward a peripheral visual stimulus (pro-saccade), or suppress that response and generate an anti-saccade in the opposite direction (Hallett, 1978). Detailed analysis of the temporal distributions of correct pro- and anti-saccades and direction errors in healthy humans and patients with neurological disease can be used to identify different signals coding response inhibition and saccade generation that contribute to the natural behavioral repertoire produced in these tasks (Coe and Munoz, 2017). In this paper we introduce a computational model of action selection, based on Trappenberg et al. (2001), that includes conceptualized inputs inspired by sensory, automated, voluntary, and inhibitory components of neural activity. This model is intended to isolate the various types of information coalescing within the primate brain to guide saccadic action selection. The goal is to improve the behavioral performance of the model (e.g., better emulate human behavior), by investigating the function of interactions within neural circuits and systems that control specific behaviors. We first provide an extended Introduction bridging three fields of systems neuroscience: human saccadic eye movement behavior, monkey neurophysiology, and computational modeling of decision making processes. We then present our new model.

### Human Saccadic Eye Movement Behavior

**Figure 1:**
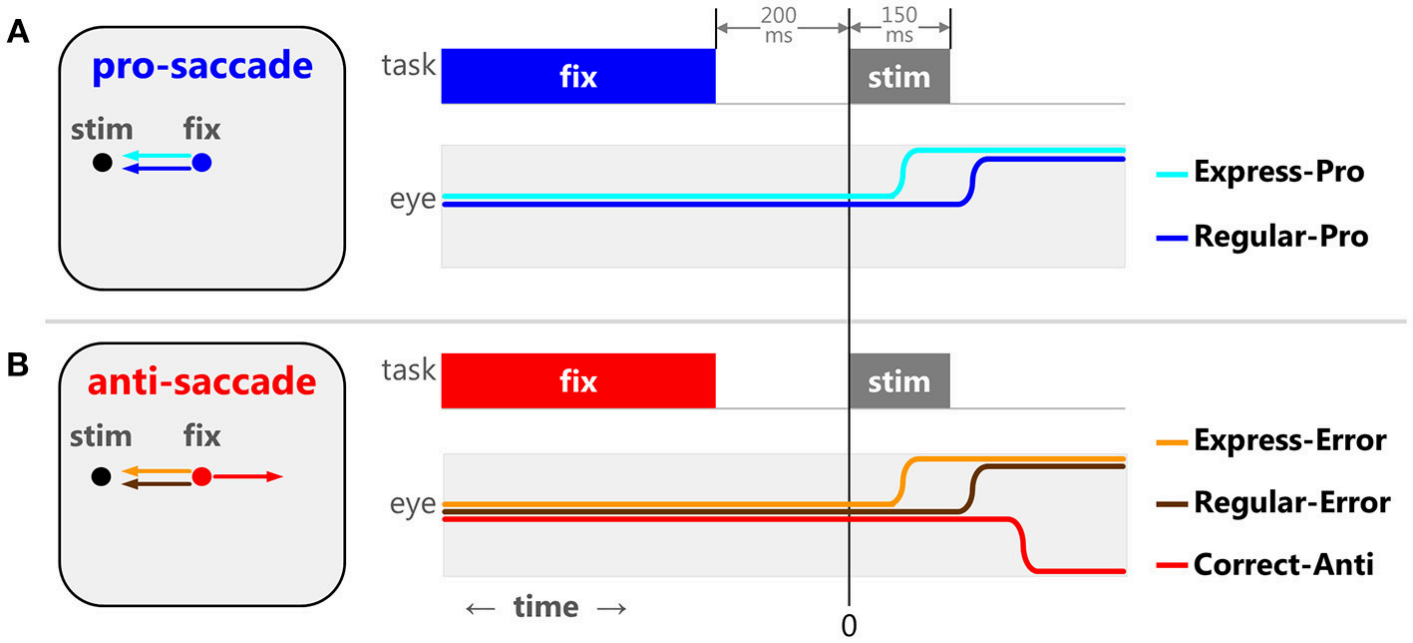
The pro- and anti-saccade tasks and the five specific types of saccades. When a 200 ms gap between the fixation point disappearance and stimulus appearance is implemented, the pro-saccade task **(A)** elicits 2 stereotypical behaviors: express latency (cyan) and regular latency (blue) correct saccades. The anti-saccade task **(B)** elicits 3 stereotypical behaviors: correct anti-saccades (red), express latency (orange), and regular latency (brown) direction errors.

Saccade tasks produce reliable behaviors that elucidate stages of cognitive maturation, aging, and the effects of neurological disorders in humans (Munoz et al., 1998; Kramer et al., 2005; Luna et al., 2008; McDowell et al., 2008; Coe and Munoz, 2017). During the pro-saccade task (Figure 1A), participants are instructed to look toward a peripheral stimulus, requiring a basic sensory-motor transformation and an automated, well-practiced, response. The distributions of saccadic reaction times (SRT) seen in this task are often bi-modal, consisting of express latency and regular latency saccades. Express saccades (Fischer and Boch, 1983; Fischer and Ramsperger, 1984) are thought to be guided by reflexive drives and pathways, as they are facilitated by introducing a predictable, temporal gap between the disappearance of the central fixation point and the appearance of the peripheral stimuli [gap effect, (Saslow, 1967; Mayfrank et al., 1986)], and require an intact Superior Colliculus (SC) (Schiller et al., 1987). Regular saccades are believed to be guided by both automated and voluntary drives. Regular latency saccades have also been described as “fast-regular” and “slow-regular” (Fischer and Weber, 1992, 1997; Fischer et al., 1993a,b). The overlapping reaction times of reflexive express, “fast-regular,” and “slow-regular” saccades can make these saccades difficult to tease apart and the arbitrary temporal delineations between them should not be considered unequivocal. In other words, not all express latency saccades are reflexive saccades. However, these three types have been manipulated experimentally using stimulus size (Ploner et al., 2004), and have been modeled statistically to show how training increases the proportion of reflexive express (Paré and Munoz, 1996) and automated “fast-regular” saccades over “slow-regular” saccades (Gezeck et al., 1997).

In the anti-saccade task (Figure 1B), participants are required to suppress the sensory-cued pro-saccade and instead perform a voluntary anti-saccade in the opposite direction. We argue that there are different types of response inhibition used to prevent express vs. regular latency direction errors (reviewed in Coe and Munoz, 2017). Pre-emptive inhibition is required to block express latency saccades to the stimulus. To prevent regular latency direction errors, the voluntary drive to make the anti-saccade must override the automated drive to make the pro-saccade. Detailed analyses of SRT and direction errors have provided significant insight into the neurophysiology behind the sensory, motor, and cognitive processes involved in action selection and response inhibition.

### Neurophysiology

**Figure 2:**
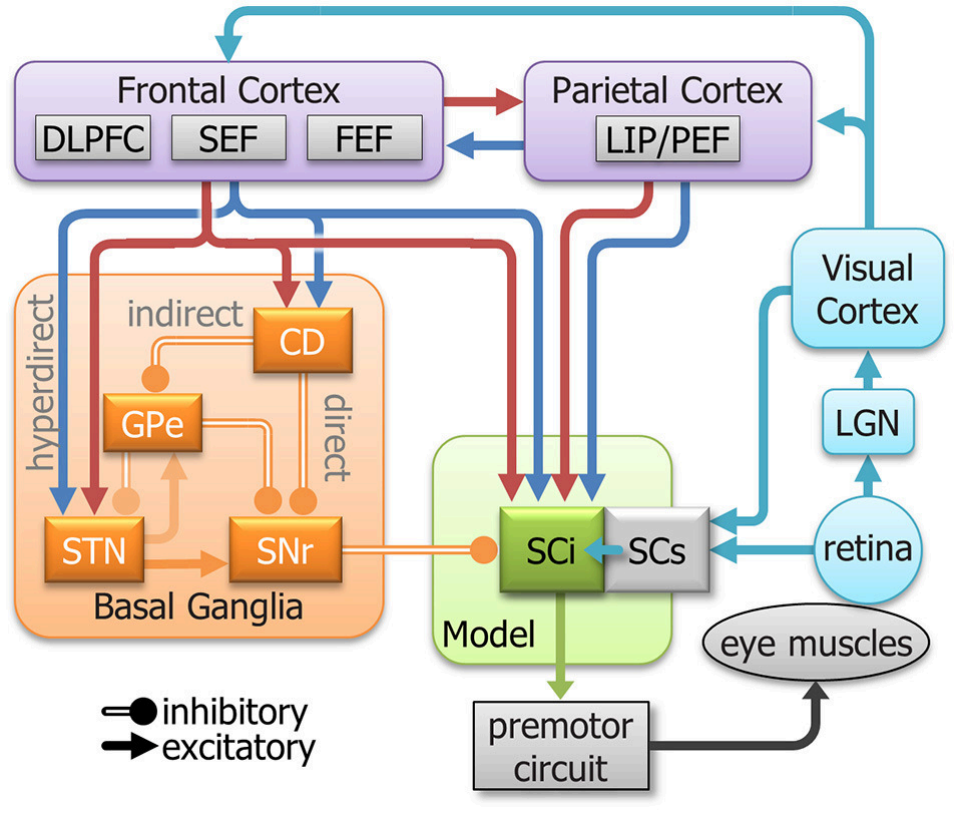
A simplified model of the oculomotor system with streamlined neural signals impinging on the SCi that influence saccades. The colors are meant to represent a highly simplified classification of the signal components that inspired the inputs to the model (See Figure 3). They do not represent all signals in each area. Cyan indicates *sensory*; Blue indicates *automated*; Red indicates *voluntary*; and orange indicates *inhibitory*. DLPFC, dorsolateral prefrontal cortex; SEF, supplementary eye fields; FEF, frontal eye fields; LIP/PEF, lateral intraparietal cortex/parietal eye fields; LGN, lateral geniculate nucleus; SCi, intermediate layers of the Superior colliculus; SCs, superficial layers of the superior colliculus; CD, caudate nucleus; GPe, external globus pallidus; STN, subthalamic nucleus; SNr, substantia nigra pars reticulata.

Eye movement neural circuitry has been studied intensively for the past 40 years. In humans, this includes saccadic behavior, functional imaging, and clinical studies. In animal studies, this includes saccadic behavior, neurophysiology, anatomy, and pharmacology. Critical sites in the oculomotor network include regions of the parietal and frontal cortices, basal ganglia, thalamus, SC, and brainstem reticular formation(Gandhi and Katnani, 2011; Krauzlis et al., 2013; Kim and Hikosaka, 2015; Schall, 2015; Hikosaka et al., 2018a). Figure 2 highlights selected areas of interest for our revised model. Instead of attempting to replicate cell types as inputs to the model, we focus on “components” of neuronal activity identified from single unit recordings in these areas. Most areas contain multiple signal components (e.g., sensory, motor, preparation, inhibition). Our goal is to emulate and modulate these components separately to see how each contributes to action selection and response inhibition. The main body of the model is based on the known circuitry of the SC, which is a central structure for saccade control (for review, see Hall and Moschovakis, 2003; Krauzlis et al., 2004, 2013; Gandhi and Katnani, 2011; White and Munoz, 2011). The SC is a multilayered structure divided into superficial layers (SCs, visual input), and intermediate/deeper layers (SCi, visual, cognitive, motor integration). The SCi has a retinotopically coded map for saccadic eye movements (Robinson, 1972) and receives signals from many structures including the SCs (Saito and Isa, 2005), posterior parietal cortex (Lynch et al., 1985; Andersen et al., 1990), frontal cortex (Segraves and Goldberg, 1987; Seltzer and Pandya, 1989), and basal ganglia (Hopkins and Niessen, 1976; Jayaraman et al., 1977; Hikosaka and Wurtz, 1983d). These numerous connections from the parietal cortex, frontal cortex, basal ganglia, and SCs make the SCi an excellent structure to study how sensory, automated, voluntary, and inhibitory signals combine, both cooperatively and competitively, to guide action selection in the pro- and anti-saccade tasks.

Sensory inputs are fashioned after the retinotectal and retino-geniculo-cortical pathways to the SCi (Moschovakis, 1996). These are the fastest routes and are believed to facilitate direct sensory-motor transformations and trigger reflexive saccades (Isa, 2002; Marino et al., 2015). Removal of the SC from the monkey leads to inability to execute express latency saccades, thereby implicating the SC in sensory driven express saccade generation (Schiller et al., 1987).

Signals from frontal and parietal cortices to the SCi carry both visual and cognitive signals (Segraves and Goldberg, 1987; Shook et al., 1991; Paré and Wurtz, 1997; Everling and Munoz, 2000; Everling and Johnston, 2013). Tonic fixation-related activity has been recorded from neurons in parietal (Lynch et al., 1977; Sakata et al., 1980) and frontal (Bon and Lucchetti, 1992; Everling and Munoz, 2000; Amador et al., 2004) cortex as well as SCi (Munoz and Wurtz, 1992, 1993a,b). This activity is both dependent (i.e., automated fixation) and independent (voluntary fixation) of a visual stimulus on the fovea. The motor responses in the frontal and parietal cortices can also be automated or voluntary. The strength of the response of neurons in the lateral intraparietal cortex (LIP) to a visual stimulus could be viewed as part of an automated motor plan (Snyder et al., 1997; Gottlieb et al., 1998; Bisley and Goldberg, 2010). Additionally, neurons in the dorsolateral prefrontal cortex (DLPFC), supplementary eye field (SEF), frontal eye field (FEF), and LIP have been shown to discharge prior to saccades in complete darkness, independent of a visual stimulus (e.g., memory-guided saccades, anti-saccades), indicating a voluntary motor plan (Goldberg and Bruce, 1990; Funahashi et al., 1993; Schlag-Rey et al., 1997; Everling and Munoz, 2000; Johnston and Everling, 2006; Stuphorn et al., 2010). In the frontal cortex, this voluntary motor command does not require a visual stimulus (Goldberg and Bruce, 1990; Schlag-Rey et al., 1997; Everling and Munoz, 2000). Together, these findings show that both the frontal and parietal cortices are strongly involved in automated and voluntary components of action selection.

The basal ganglia are also an integral part of the neural circuitry for action selection. The Caudate Nucleus (CD) receives the main input to the basal ganglia from cerebral cortex and provides input via the direct and indirect pathways to the Substantia Nigra pars Reticulata (SNr). The SNr is part of the output for the basal ganglia and projects directly to the SCi (Hikosaka and Wurtz, 1983d) via an inhibitory synapse (Vincent et al., 1978; Di Chiara et al., 1979; Chevalier et al., 1981). Here, we focus on two types of inhibitory processes seen in the basal ganglia. The first process can be described as an inhibitory gate, where tonic inhibition is removed from a specific location at the time of a motor action. This type of location-specific dis-inhibition has been shown in the Substantia Nigra pars Reticulata (SNr) (Hikosaka and Wurtz, 1983a,b,c; Handel and Glimcher, 1999). The other inhibitory process is global and holds back all movement, like a parking break. When it releases the entire peripheral field is dis-inhibited allowing excitatory activity to propagate through the SCi. This type of activity has been observed in the “omni-directional” pause neurons (Kato and Hikosaka, 1995) in the external globus pallidus (GPe) and “universal pausers” (Handel and Glimcher, 1999) in the SNr.

These numerous and complicated inputs to the SCi coalesce, both cooperatively and competitively, to guide saccadic eye movements. It is components of these signals that inspired the inputs for our computational modeling of decision making processes.

### Computational Modeling of Decision Making Processes

The expansive knowledge of the oculomotor system has helped build a solid foundation for modeling saccade behavior. Using information about the brainstem circuitry and the on-line control of individual saccades led to models of the saccade burst generator and the SC (for a review see Girard and Berthoz, 2005). Early models of saccadic behavior primarily focused on producing the correct kinematics for saccade generation (Robinson, 1975; Jürgens et al., 1981; Quaia et al., 1999) and the variable shape of individual SRT distributions (Carpenter and Williams, 1995). More recently, models that employed neural fields (Amari, 1977), were fundamental in decision-making models that concentrated on the competitive interactions between visually driven and cognitively driven inputs to vie for action selection (Carpenter and Williams, 1995; Kopecz, 1995; Trappenberg et al., 2001; Camalier et al., 2007; Cutsuridis et al., 2007; Schall et al., 2011).

The LATER model (Carpenter and Williams, 1995) used trial-by-trial variation in the linear rise of decision signals that needed to cross a fixed threshold to emulate SRT variability (see review Noorani and Carpenter, 2016). Neural correlates of decision-making via the accumulation of information have been found in numerous areas of the brain (Kim and Shadlen, 1999; Shadlen and Newsome, 2001; Heekeren et al., 2004; Huk and Shadlen, 2005; Gold and Shadlen, 2007; Ding and Gold, 2010, 2012; Mante et al., 2013). Subsequent iterations of the LATER model were designed for the countermanding task (Logan and Cowan, 1984) and included a “go” and a “stop” input to estimate stop signal reaction times (Hanes and Carpenter, 1999). These models were quite elegant in their simplicity and explanation of SRT distributions. However, each “decision” input (e.g., “go” or “stop”) directly represented one specific behavioral outcome and they did not interact with each other. The LATER model has been redeveloped in several ways to address anti-saccade and countermanding tasks (Noorani and Carpenter, 2013; Noorani, 2014), and inhibition and error rates in anti-saccades (Aponte et al., 2017, 2018a,b).

Kopecz and Schoener (Kopecz, 1995; Kopecz and Schöner, 1995) introduced distinctive “visual” and “intentional” inputs that represented two potentially competing behavioral goals. These inputs interacted in a 1-dimensional, laterally-inhibitory, neural field (Amari, 1977). The progression here was to move from abstract decision signals, which were independent, to inputs that interacted and could inhibit each other. The two Gaussian-like inputs (representing activity across horizontal space) were locally excitatory and distally inhibitory to represent the inhibitory interplay within the saccadic system between two mutually exclusive eye movement commands. The pattern local-excitation and distal-inhibition is quite robust and has been noted in sensory cortex (Grossberg, 1973; Wilson and Cowan, 1973), the basal ganglia (see Nambu, 2008 for review) and the SC Meredith and Ramoa, 1998; Munoz and Istvan, 1998; Olivier et al., 1999; Munoz and Fecteau, 2002; Phongphanphanee et al., 2014. Importantly, for this type of model is that the variability of the SRT distributions was based on the interaction of both inputs in a neural field. Importantly, the strength of the “losing” input could still affect SRT. Usher and McClelland (2001) compared their “leaky competing accumulator model” with existing diffusion, random walk, and accumulator models to show how models similar to Kopecz and Schoener's were able to address a wider range of data from perceptual choice experiments. In addition, they found that the neural field model described the nonlinear integrations during sensory motor transformations better than linear models.

Trappenberg et al. (2001) introduced a model designed to emulate SCi activity during several oculomotor tasks, including the anti-saccade task. Similar to the Kopecz model Kopecz (1995), there were two inputs racing to a fixed threshold: an “endogenous” input that represented the intended location of the anti-saccade, and an “exogenous” input that represented the location of a visual stimulus. Again, these inputs were potentially mutually inhibitory and competed in a 1-dimensional neural field that used competitive integration. This model used neural recordings of SC activities to guide the shape of the inputs and their integration in the model. Behavioral results from this model were impressive because they simulated the bimodality of pro-SRTs, and anti-SRTs were slower. Although they addressed the possible sources of express latency saccades, they did not address direction errors. Recent work by Bompas and Sumner (2011) directly compared an extended version of the LATER model and an extended version of the Trappenberg model with a focus on the effects of transient visual information on saccade behavior. They found that their updated Trappenberg model could account for inhibitory effects of distractors on SRT, while their updated LATER model could not. This was primarily due to the interactive nature of inputs in a dynamic neural field model. Additionally, spatial interactions of multiple sensory inputs using dynamic fields has been examined in a 2-dimensional version of the Trappenberg model (Marino et al., 2012) that explained SRT changes based on stimulus intensity, as well as the number and location of stimuli. Cutsuridis et al. (2007) created another rendition of a dynamic neural field model that used even more types of “cells,” including fixation cells, further improving behavior. Their model was able to produce direction errors but did not perform express latency saccades for either pro-saccades or direction errors. The common theme from these papers is that the authors used neural recordings to constrain the size and shape of the model inputs, and improved performance.

Saccade initiation or decision models can clearly be formulated in numerous different ways, but our previous model lent itself better to the integration of relatively simple inputs that would interact, spatial and temporally, and could easily be manipulated. We reconceptualized the Trappenberg model with neurophysiologically inspired inputs, but instead of cell types, we used components of signals that have been recorded in areas of the primate brain during behavioral experiments, aiming for a systems-neuroscience approach. For the current model, 8 inputs were designed to emulate components of sensory, automated, voluntary, and inhibitory signals known to impinge on the SCi. Admittedly, this strategy complicates a well-functioning model that used only two inputs (Trappenberg et al., 2001), but we were confident that a more detailed set of inputs would be capable of producing a wider range of normal saccadic behavior. Implementing inputs based on components of neural activity allow us to test for causal links between each input and the behaviors the model simulates.

## Materials and Methods

### Behavior and Nomenclature

The pro- and anti-saccade tasks (Figure 1) elicit several stereotypical behaviors in humans (Fischer and Weber, 1992; Fischer et al., 1993b; Munoz et al., 1998; Peltsch et al., 2011; Coe and Munoz, 2017). The pro-saccade task (Figure 1A) generally features a range of SRT distributions for correct saccades, including express and regular latency saccades (Fischer and Boch, 1983; Fischer and Ramsperger, 1984). The exact timing of express latency saccades depends on lab conditions and variability across participants, but is generally considered to be around 90–140 ms after a stimulus appearance for healthy adult humans. Saccades after this window are referred to as regular latency saccades. The anti-saccade task (Figure 1B) represents a competition between a desirable voluntary anti-saccade to an abstract location and undesirable sensory-cued saccade toward the peripheral stimulus. This competition can either delay the voluntary anti-saccade or sometimes the automated command can override it, resulting in direction errors. During the anti-saccade task, visually triggered saccades result in direction errors that have express latency reaction times. Similar to the pro-saccades, we separated express and regular latency anti-saccade direction errors. The frequency of these two types of direction errors (express and regular latency) changes through development, maturation, and aging and is indicative of different mechanisms of saccadic production and response inhibition (Coe and Munoz, 2017). Thus, we have formalized five specific types of saccadic behaviors observed in the pro- and anti-saccade tasks based on SRT and the saccade selected:

1. Regular latency correct pro-saccades (Regular-Pro)
2. Express latency correct pro-saccades (Express-Pro)
3. All correct anti-saccades (Correct-Anti)
4. Regular latency anti-saccade direction errors (Regular-Errors)
5. Express latency anti-saccade direction errors (Express-Errors)

These five types of saccades reveal distinct aspects of sensory, automated, voluntary, and inhibitory signals in the saccadic eye movement system. For the purpose of this model, *Sensory* signals are entirely driven by environmental stimulus properties. *Automated* signals are initiated by an external stimulus but can trigger a pre-determined neurological circuit (like a domino effect), honed through previous experience and training, to propagate a well-practiced motor command. *Voluntary* signals are initiated and sustained by internal drives and are not reliant on external or environmental stimuli. Finally, *Inhibitory* signals are tonic holding commands that must be removed and/or overcome in order to execute motor commands. During the pro- or anti-saccade task, the activity of an individual task-related neuron in the oculomotor network may display one or more of these signals. We recapitulated these neural signals as individual inputs to our model. To help clarify, the term “signals” will be used to described specific components of neuronal activity, and the term “inputs” will be used to described the inputs created for the model. If we simulate neural signals properly, using individual inputs, then the output of the model (e.g., SRT, and saccade selection) should show similar patterns to human behavioral data. The use of multiple component-based inputs allows for specific causal links to be drawn between these five types of saccades and the underlying neural processes that produce them.

### The Neural Field Model

**Figure 3:**
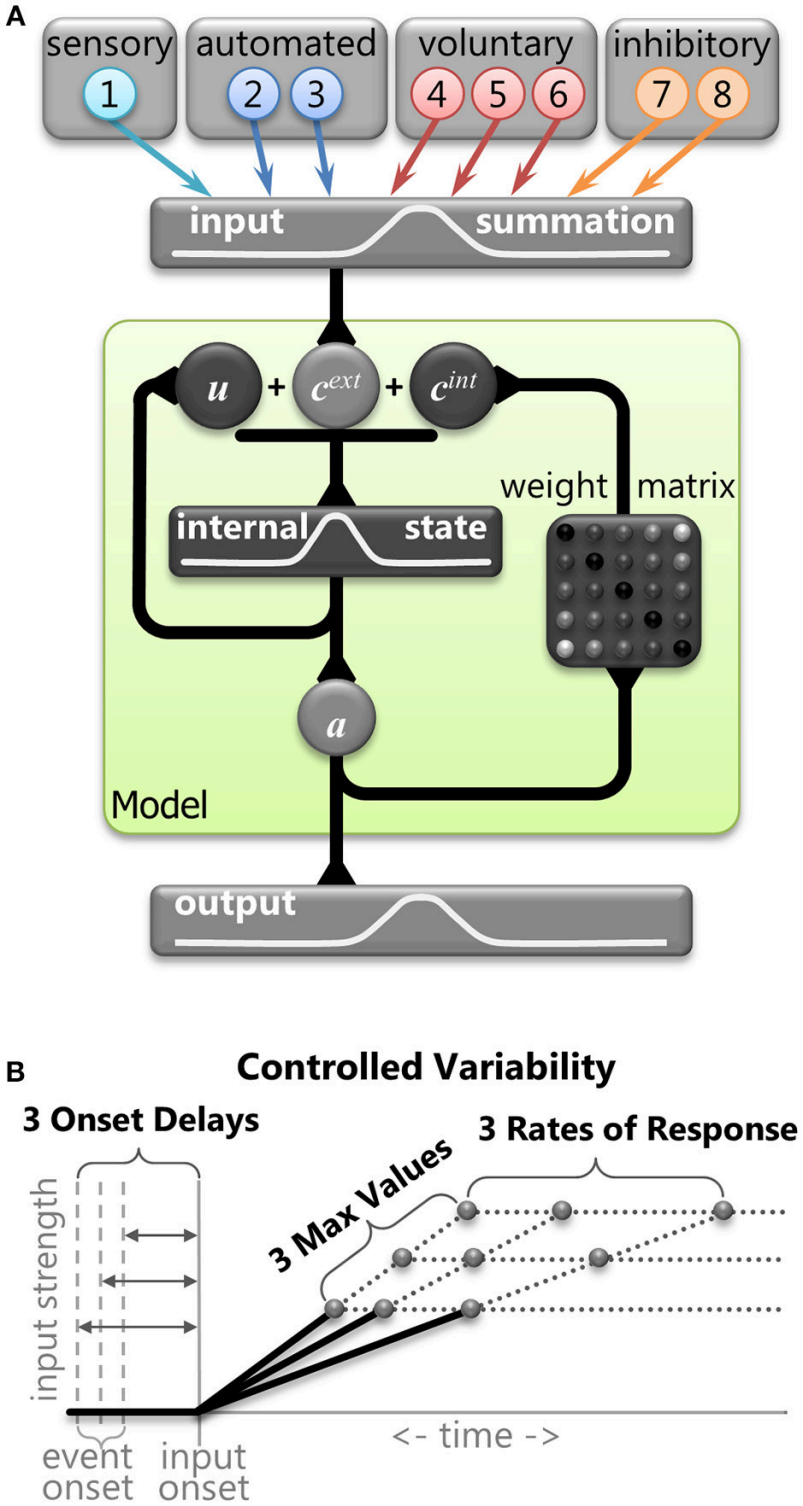
**(A)** Graphical representation of the mathematical model. The primary stage shows the eight inputs that were derived from components of neural signals in the brain. The central stage (within the green shaded box) demonstrates the dynamic internal state of the model. Vector c^ext^ represents the combination of the external inputs. Vector c^int^ represents the internal connections across the model (Equation 6). Vector *u* represents internal state of the model (equation 5). Vector *a* represents output activity from the model (Equation 3). The final stage is the output activity. **(B)** A graphical representation of the three levels of controlled variability for the Onset Delay, the RoR, and the MaxVal for various inputs (see Table 1).

**Figure 4:**
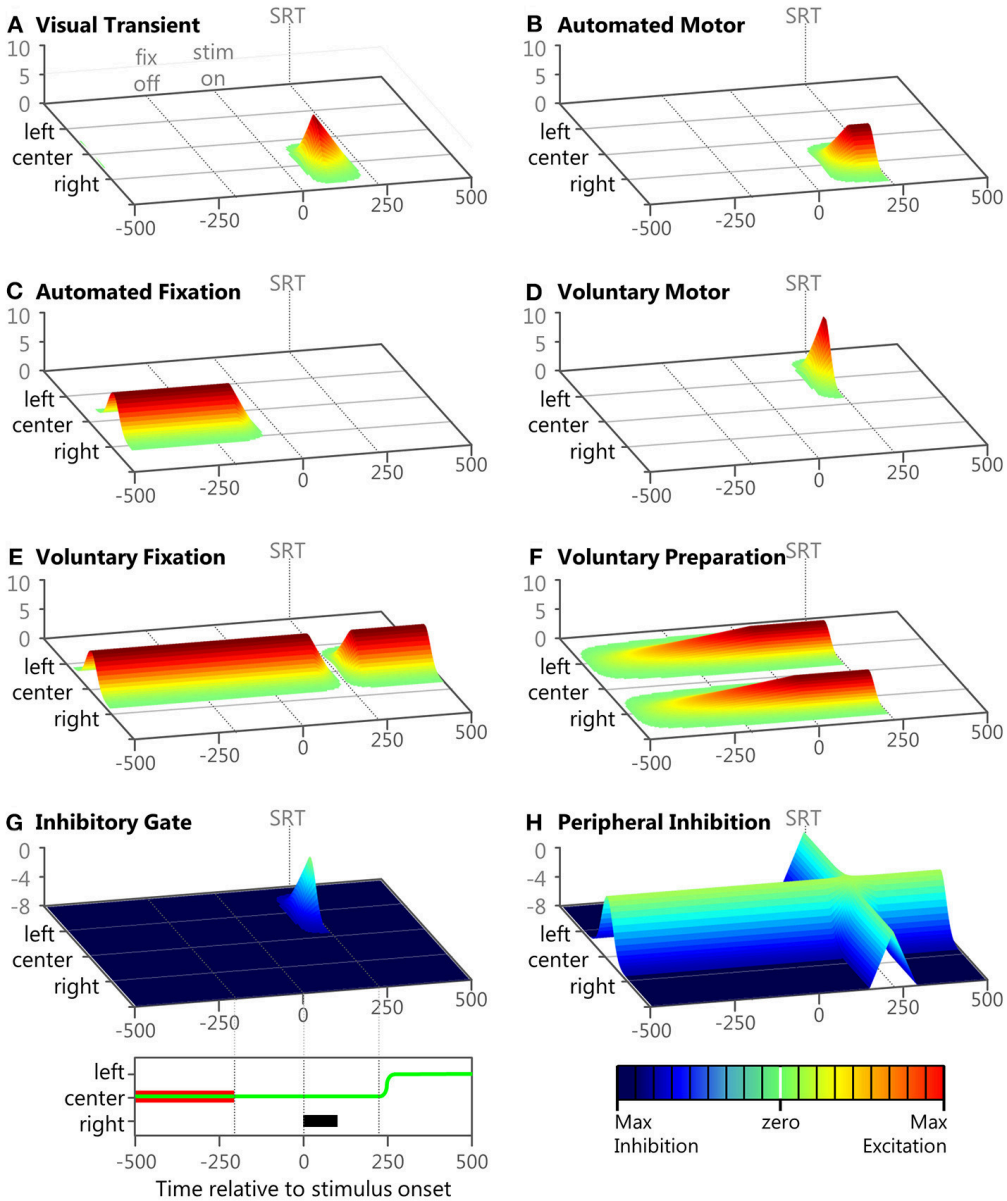
Examples of the eight inputs during a correct anti-saccade trial. Panels **(A–H)** show 3D plots of intensity (bottom right legend) over time (bottom left legend) and space for the 8 inputs during an example trial described in the Methods section. The time scale of each panel starts after the model has fixated on the central fixation point. Each panel represents the model's visual space across 100 nodes. The fixation point is presented at the 50th node labeled “center,” the peripheral stimulus is presented at the 75th node labeled “right,” and the anti-saccade goal is the 25th node labeled “left.” At the bottom left is a panel showing *task space* where the anti-saccade direction is to the left, the fixation is in the middle and the stimulus location is to the right. The fixation point is indicated by the red bar, the peripheral stimulus is indicated by the black bar, and the eye-position is indicated by the green line. At the bottom left is a color map to indicate input strength (in arbitrary units). In each panel, fixation offset is the earliest dotted line (−200 ms), stimulus onset is the middle dotted line (0 ms), and saccade initiation is indicated by the final dotted line.

The basic assumptions and framework of the model and inputs were based on the Trappenberg et al. (2001) model. It used competitive integration of an exogenous input and an endogenous input in a laterally inhibitory neural field intended to emulate neural dynamics within the SCi. We used this type of model to simulate horizontal eye movements and hence employed a 1D-feature space defined by nodes that represents horizontal visual space and represents 10 mm of the SCi bilaterally. We used a discretization of the fields with a vector of 100 nodes (*N* = 100) to implement the feature space from −5 mm (far left) to 5 mm (far right), where zero indicated the center. This discretization was the same for the input field (Figure 3A, top), the field governed by competitive integration dynamics (Figure 3A, middle), and the output activity (Figure 3A, bottom). First, we describe the 8 inputs with the help of samples from a correct anti-saccade trial (Figure 4) so we can explain the theory and implementation. We then describe the competitive integration dynamics of the neural field model itself (Figure 3A), and finally we describe the controlled variability applied to the inputs (Figure 3B).

#### The Eight Component-Based Inputs

We implemented 8 inputs, which were manipulated separately. These inputs emulated components of neural activity identified in the primate brain during the performance of saccade tasks. Although these inputs were inspired by the numerous signals converging onto the SCi, they were not intended to represent actual firing rates of particular neurons in specific brain areas. Rather, they represent individual components of neural activity, often seen in multiple brain areas. All 8 input vectors were updated at each time point (Equation 1) and were defined by the following attributes: an Onset Delay, a linear change in strength characterized by a rate of response (RoR), a maximum value for its strength (MaxVal), and the center of activity (μ). The settings for some of these attributes were varied deterministically to add structured variability, and are described in more detail below. The Onset Delay was the amount of time between an event and the onset of the RoR for that input. Whereas previous accumulation models (Carpenter and Williams, 1995; Trappenberg et al., 2001) discussed only rate of rise for the inputs, here the term Rate of Response is used because the inputs could weaken (where values move toward zero) as well as strengthen (where values move away from zero either negatively or positively). The MaxVal was the absolute value of how far from zero an input could get. The RoR was a proportion of the fixed spatial function *k* (Equation 2) that was added to or subtracted from the input's current value to create the input's updated value. Generally, lower-case bold letters indicate vectors and upper-case bold letters denote matrices.

(1) $$\begin{array}{r} {input}_{i}\left(\;t+\Delta t\right)={input}_{i}\;\left(t\right)\;+\;\left(k_{i}^{\mu }\times RoR\times \Delta t\right), \end{array}$$

The node index *(i)* indicates position in space (i.e., 1:100) and Δ*t* indicates the increment of time (Δ*t* = 1 ms for all simulations). The value of **k** at each node depended on the node's smallest distance from the center of activity (μ) according to a Gaussian profile with standard deviation (γ) of 0.6 and an amplitude (*amp*) of 1.05, following closely the values from the original Trappenberg et al. (2001) model settings.

(2) $$\begin{array}{r} k_{i}^{\mu }=amp\times exp\;\left(\frac{-{\left(\min\left(\left|i\Delta x-\mu |,\;N-|i\Delta x-\mu \right|\right)\right)}^{2}}{2\gamma^{2}}\right). \end{array}$$

The numerator in the exponential is the minimal distance of a node to the center of the Gaussian when representing the feature space on a torus (ring) to avoid boundary conditions. This is a common computational technique with neural fields. Examples of the eight inputs during a sample correct anti-saccade trial, with the visual stimuli on the right (μ = 2.5) and the anti-saccadic goal on the left (μ = −2.5), are presented in Figure 4 to illustrate their temporal and spatial nature. This trial's specific Onset Delay, RoR, MaxVal, and μ for each of the eight inputs during this example trial are given in the text.

##### Visual transient

**Input #1** (Figure 4A; Onset Delay = 50 ms; RoR = 15%; MaxVal = 8; μ = 2.5). This sensory input had a brief burst soon after the onset of a visual stimulus. It was modeled after components of activity seen in the magnocellular LGN (McAlonan et al., 2008) and the superficial layers of the SC (Boehnke et al., 2011; Marino et al., 2012). Once the model had fixated on the central fixation point the visual scene remained constant so the *Visual Transient* input returned to zero across the entire field. When the visual stimulus appeared in the right periphery of visual space, plus a 50 ms afferent delay (White et al., 2017), there was a brief response of the *Visual Transient* input on the right side of the model's feature space. Thus, the *Visual Transient* input increased at each iteration by ***k***^2.5^ × 0.15 × 1 (Equation 1) until it rose to its MaxVal. The *Visual Transient* started to fade using half its RoR 50 ms after its response onset and decreased until it was zero across the entire field. The *Visual Transient* input was also reset to zero after each saccade.

##### Automated motor

**Input #2** (Figure 4B; Onset Delay = 60 ms; RoR = 6%; MaxVal = 6; μ = 2.5). This input represented an externally triggered, but self-propagating motor command to drive a saccade to a peripheral visual stimulus. The timing of this input was derived from recordings in macaques (Barash et al., 1991; Duhamel et al., 1992; Gottlieb and Goldberg, 1999) and has been used in previous models (Noorani and Carpenter, 2013; Noorani, 2014) for similar stimulus driven inputs. We modeled response activity that did not necessarily elicit a motor command, similar to where the strength of the initial response of some neurons was not related to whether or not a saccade was made to a peripheral stimuli (Snyder et al., 1997; Gottlieb et al., 1998; Bisley and Goldberg, 2010). As illustrated in the example trial, the location of peak activity (μ in equation 2) for the *Automated Motor* Input was always at the spatial location of the visual stimulus. We gave automated inputs a fixed Onset Delay because they were triggered externally, but the RoR and MaxVal were one of three settings (Table 1), representing different levels of automation. The *Automated Motor* input stayed at its trial-specific MaxVal until a saccade was made, after which it was reset to zero.

**Table 1:** The possible settings used for the attributes of each input for each trial.

**Inputs**	**Onset delay (ms)**	**Rate of response (%)**	**Maximum value**
(1) Visual transient	50	15	8
(2) Automated motor	60	4, 6, 8	4, 6, 8
(3) Automated fixation	60	10	6
(4) Voluntary motor	140,155,170^*^	5, 10, 15	Dependent
(5) Voluntary fixation		10	4, 6, 8
(6) Voluntary preparation		Dependent	4, 6, 8
(7) Inhibitory gate		5, 10, 15	4, 6, 8
(8) Peripheral inhibition		5, 10, 15	4, 6, 8

* *Inputs 4–8 had the same onset delay per trial*.

*only 10 attributes had 3 possible values for both the pro- and anti-saccade tasks leading to 118,098 (3^10^ × 2) possible trials. The Voluntary Motor MaXVal was dependent on when the saccade was made and the Voluntary Preparation RoR was dependent on its MaxVal*.

##### Automated fixation

**Input #3** (Figure 4C; Onset Delay = 60 ms; RoR = 10%; MaxVal = 6; μ = 0.0). This input also represented an externally triggered excitatory component, but only for a visual stimulus that was currently foveated, and was modeled after components of activity seen in the parietal cortex (Lynch et al., 1977) related to fixation control. Similar inputs with similar timing have been used before (Heinzle et al., 2007). In the example trial illustrated in Figure 4C, the fixation point was already illuminated and the model was fixating on it so the *Automated Fixation* input was at MaxVal at the center. After the fixation point disappeared to start the gap period, plus the fixed Onset Delay, the *Automated Fixation* input started to fade using its negative RoR. As this trial produced a regular latency saccade, there was no activity from the *Automated Fixation* input for the stimulus because the saccade occurred after the stimulus disappeared.

##### Voluntary motor

**Input #4** (Figure 4D; Onset Delay = 170 ms; RoR = 15%; μ = –2.5). This input represented an internally initiated motor command that did not require a visual stimulus and was modeled after components of activity seen in the frontal cortex (Goldberg and Bruce, 1990; Schlag-Rey et al., 1997; Everling and Munoz, 2000). This type of signal has been argued to be specifically important for non-visually driven saccade performance (Funahashi et al., 1993; Johnston and Everling, 2006). Because this input was fashioned after a voluntarily initiated motor command, it had a longer Onset Delay than the automated inputs. Also, it was not initiated by stimulus appearance, so it had a variable Onset Delay as well as RoR. The earliest Onset Delay setting was chosen to be after the express latency window (at 140 ms) and we arbitrarily chose 15 ms increments for the medium and late settings (Table 1). All voluntary inputs (4–8) had the same Onset Delay for a given trial. Admittedly, defining the onset timing for voluntary inputs unbound to external triggers is a vague concept but the precise values we used are trivial. Here, we merely assert that voluntary signals take longer to start than automated signals and that their onset timing is more variable. The spatial peak of this input was centered on the saccadic goal as defined by the task, not necessarily the stimulus. During a pro-saccade trial the peaks of the *Automated Motor* and the *Voluntary-Motor* inputs were spatially aligned on the visual stimulus so they were cooperative, but during an anti-saccade trial the peaks were on diametrically opposed sides of space (Figures 4B,D) supporting mutually exclusive saccades in opposite directions.

In our example anti-saccade trial, the *Voluntary-Motor* input (Figure 4D) started at zero strength across the neural field and after the visual stimulus onset, plus the medium Onset Delay, it built up strength (determined by the RoR) at the location of the saccadic goal on the left (i.e., ***k***^−2.5^ × 0.15 × 1), until a saccade was executed. Due to the goal-driven nature of this signal, the *Voluntary-Motor* input was allowed to rise until a saccade was initiated. A low MaxVal would merely represent that the “participant” does not want to perform the task. We presume that the goal is to participate so there was no value in limiting the Voluntary-Motor. After a saccade was executed the *Voluntary-Motor* input was reset to zero.

##### Voluntary fixation

**Input #5** (Figure 4E; Onset Delay = 170 ms; RoR = 15%; MaxVal = 6; μ = 0.0). This input represented an internally initiated command to maintain fixation, even without a visual stimulus present, and was modeled after components of fixation activity in the frontal cortex (Sakata et al., 1980; Bon and Lucchetti, 1992; Amador et al., 2004). Clear correlates also exist in the SCi itself (Munoz and Wurtz, 1993a; Everling et al., 1999). In the example anti-saccade trial, the model was already fixating so the *Voluntary Fixation* input was already at its MaxVal and was centered on the fovea. In unison with the onset of the *Voluntary-Motor* input, the *Voluntary Fixation* input decayed using a negative RoR until it reached zero or until a saccade was initiated. Once a saccade was made, this input returned to its peak strength using a positive RoR.

##### Voluntary preparation

**Input #6** (Figure 4F; Onset Delay = 170 ms; MaxVal = 4; μ = –2.5 and 2.5). This input represented an internally initiated preparatory signal that does not require a visual stimulus and was modeled after components of activity observed in the SEF and FEF (Schlag-Rey et al., 1997; Everling and Munoz, 2000; Coe et al., 2002). The Onset Delay for this input was relative to when fixation was established on the central cue. The model was imparted with the “knowledge” of when the stimulus would appear and that the stimulus could appear at one of two possible locations. We modeled this information by using a two-peaked excitatory input (e.g., max [***k***^−2.5^, ***k***^**+**^^2.5^]) that slowly built up to reach its MaxVal at the expected stimulus onset time. This type of activity has been directly measured in the SCi using different fixation durations (Thevarajah et al., 2009). In the example anti-saccade trial (Figure 4F), once fixation was established, and after the Onset Delay, the *Voluntary Preparation* input began to rise at a rate that was dependent on its MaxVal. We gave this input a dependent RoR to ensure that this input's two peaks reached the MaxVal at the predictable onset of the visual stimulus. It sustained that value until a saccade was made. After a saccade was executed the *Voluntary Preparation* input was reset to zero.

##### Inhibitory gate

**Input #7** (Figure 4G; Onset Delay = 170 ms; RoR = 10%; MaxVal = 8; μ = –2.5). This input was designed to provide a wall of inhibition across the entire visual field holding back motor commands. When a specific motor command was ready only a portion of the wall “opened” (like a small gate). In this fashion, the *Inhibitory Gate* input released the specific motor command by removing the inhibition at that precise section of the visual field. This type of location-specific dis-inhibition has been shown in the SNr (Hikosaka and Wurtz, 1983a,b,c; Handel and Glimcher, 1999). This input was fashioned with its MaxVal as a measure of baseline inhibitory strength and was negative, and a positive RoR would bring the inhibition to zero at a specific location. At the start of the example anti-saccade trial (Figure 4G), the *Inhibitory Gate* input was already at its maximum inhibitory strength across the entire visual field. In unison with the variable Onset Delay of the *Voluntary-Motor* input, the *Inhibitory Gate* input started to dis-inhibit the location that represented the voluntary saccade goal on the left (i.e., ***k***^−2.5^ × 0.10 × 1). In this example trial, the dis-inhibition continued until there was zero inhibition at that location or a saccade was made. Once a saccade was initiated, it returned to its maximum inhibitory strength using a negative RoR.

##### Peripheral inhibition

**Input #8** (Figure 4H; Onset Delay = 170 ms; RoR = 10%; MaxVal = 8; μ = *0.0*). This input was designed to provide inhibition in the periphery, helping to maintain fixation. When a motor command was ready, the peripheral neural field was dis-inhibited, like the release of a parking brake, allowing any excitatory activity to propagate through the SCi. This type of activity has been observed in the “omni-directional” pause neurons (Kato and Hikosaka, 1995) and “universal pausers” (Handel and Glimcher, 1999) in the basal ganglia. In our example anti-saccade trial (Figure 4H), the *Peripheral Inhibition* input started at its maximum inhibitory firing rate across the peripheral visual field and suppressed everything except fixation activity in the center of the neural field. In unison with the variable Onset Delay of the *Voluntary Fixation* input, the *Peripheral Inhibition* input releases its inhibition across the entire visual field using a positive RoR. In this example trial, the dis-inhibition progressed until there was zero inhibition across the peripheral visual field or a saccade was made. Once a saccade was made, the *Peripheral Inhibition* input returns to its maximum inhibitory strength using a negative RoR.

#### Competitive Integration Dynamics

The model's three stages are illustrated in Figure 3A. The input stage represents the sum of the 8 inputs described above (Figure 3A, top). The second stage represents the internal state of the integration nodes (Figure 3A, center). The final stage represents the output of the model (Figure 3A, bottom). The output activity across the neural field model was represented by the vector ***a***, and was calculated for each time point (*t*) as a nonlinear function of its internal state (***u***) using a sigmoidal function:

(3) $$\begin{array}{r} a\left(t\right)=\frac{1}{1+exp\left(-\beta u\;\left(t\right)\right)} \end{array}$$

where β defined the steepness of the sigmoid. This was set as 0.09 for all simulations. Larger values for β force the output activity of each node closer to a binary on-off state so smaller values are more appropriate. The Trappenberg et al. model Trappenberg et al. (2001) used 0.07 to imitate build-up neurons specifically and only a mild increase was chosen here to simulate a larger variety of neuronal firing patterns. We implemented a fixed threshold for saccade initiation by stating that when the output activity at any non-central location reached 0.7, a saccade would be made to that location. The continuous form, in both time and space, of the equation for the dynamics of the neural field (*u*) was similar to Trappenberg et al. (2001) which has been elaborated upon more recently (Trappenberg, 2010);

(4) $$\begin{array}{r} \tau \frac{du\;\left(x,t\right)}{dt}=-u\left(x,t\right)\;+\;\left({c^{ext}\left(x,t\right)+c}^{int}\left(x,t\right)\right). \end{array}$$

We used the Euler method to implement a temporally discrete version of Equation (4) for this simulation, and space was discretized by building the model using vectors of nodes:

(5) $$\begin{array}{r} u_{i}\left(t+\Delta t\right)=\left(1-\frac{\Delta t}{\tau }\right){\times u}_{i}\left(t\right)\;+\;\frac{\Delta t}{\tau }\times \left({c_{i}}^{ext}\left(t\right)\;+{c_{i}}^{int}\left(t\right)\;\right), \end{array}$$

where Δ*t* is a step parameter for the time discretization (1 ms), *i* = 1…, N is an index for the spatial location (*N* = 100), Tau (τ) is a time scale constant (set to 4 for all simulations), the external contribution (**c**^*ext*^*)* is a vector representing the contribution of the sum of the eight external inputs to the model, and the internal contribution **(c**^*int*^) is a vector representing the contribution from the internal connections across the model (Equation 6).

(6) $$\begin{array}{rcl} c^{int}\left(t\right) & = & W\times a\left(t\right) \end{array}$$

(7) $$\begin{array}{rcl} W & = & \left(G-m\right)\times \Delta x \end{array}$$

(8) $$\begin{array}{rcl} G_{i,j} & = & sf\times exp\;\left(\frac{-{\;\left(\text{ min}\left(\left|i-j|,\;N-|i-j\right|\right)\times \Delta \;x\;\right)}^{2}}{2\sigma^{2}}\right) \end{array}$$

The internal contribution (***c***^*int*^) is the matrix product of the current activity of the model (***a***) and a laterally-inhibitory weight matrix (***W****;* Equation 7).The weight matrix ***W***(equation 7) is positive (i.e., excitatory) for proximal nodes and negative (i.e., inhibitory) for distal nodes. It was created by the Gaussian matrix ***G***(Equation 8) that has been shifted by 80% of its maximum value [*m* = 0.8 × max(*G*)] and scaled by Δ*x*. The constant Δ*x* = 10/*N* represents the discrete distance in feature space between the nodes of the model from the discretization of the feature space in the range of –5 to 5 mm of SC tissue. The weight matrix ***W***was kept periodic by using the minimum circumferential distance between *i* and *j* for the Gaussian ***G*** (Equation 8), and the width of the Gaussian was set to σ = 0.85 with a scaling factor (*sf*) of 74.7.

#### Controlled Deterministic Variability

It is important to note that none of the equations above contain any random variables. In order to test the robustness of the model and to vary its behavior we introduced controlled variability by implementing three values (small, medium, or large) for the Onset Delay, RoR, and MaxVal for some of the input vectors (Figure 3B). This controlled variability of the input attributes is the only parameter that was used to create variations in SRT distributions and selection behavior. By using only three values, we could perform controlled *post-hoc* analyses on the finite number of possible outcomes to determine which settings influenced which behaviors. The three values were monotonically increasing (e.g., 4, 6, 8 or 5, 10, 15). We did not bias the frequency of any settings to attempt to force the model's behavior to match a prescribed data set; we simply wanted to show how the model's performance is modified by an equally distributed range of inputs. The actual values for the attributes of each input were kept as simple (and similar) as possible while still producing a reasonable likeness to human behavior.

The small, medium, and large values for the attributes (Onset Delay, RoR, MaxVal) gave the model a huge number of possible trials. With three values for all 24 attributes (three attributes of all eight inputs), for both tasks, we would have 3^24^ × 2 = 564,859,072,962 possible combinations; each one with its own trial and outcome. However, to focus the model on action selection, response inhibition, and reaction time variability, only 10 attributes were varied (see Table 1). As we were not interested in sensory processing variability, all attributes for the *Visual Transient* input and the Onset Delay for the automated inputs were fixed for all trials. This ensured that all trials had the same external stimulation and that all behaviors were due to factors internal to the model and not variations in the environment or sensory processing. Unlike the first 3 inputs, the voluntary and inhibitory inputs (#4–8) were initiated by internal drives that were not time-locked to any external event. We chose to implement a common internal drive for the voluntary and inhibitory inputs by varying the Onset Delays for these inputs together on a trial-by-trial basis. In preliminary work, the variation of the RoR and MaxVal for the *Automated Fixation* input only influenced the gap-effect (Saslow, 1967; Dorris and Munoz, 1995) and contributed to differences in express latency saccades between the gap task, and a step task (not shown). Similar techniques have been implemented previously to model aspects of the gap-effect (Kopecz, 1995). Additionally, the gap-effect is strongly guided by factors external to the model that we were not currently exploring. Instead of removing it, the attributes for it were fixed to singular values. In preliminary work (Coe et al., 2010) variation of the RoR (e.g., 5 to 15%) of the *Voluntary Fixation* input did not contribute to behavioral variability so it was fixed to a single value. However, the manipulation of the MaxVal for the *Voluntary Fixation* input did contribute to behavioral variability and remained in the model. The build-up activity seen in the SC has been shown to be independent of the duration of the pre-stimulus window and that it is the final level of build-up activity that is important in both the SC (Thevarajah et al., 2009) and SEF (Coe et al., 2002). Thus, the RoR of the *Voluntary Preparation* input was dependent on its varying MaxVal and was equal for both left and right stimulus locations, emulating a temporal expectation that was not biased to either direction. Due to the goal-driven nature of *Voluntary-Motor* input, this input had no maximum value and would continue to rise and compete until a saccade was made. Thus, in total there were 3 values for ten attributes in both the pro- and anti-saccade tasks creating 3^10^ × 2 = 118,098 combinations, or trials (see Table 1).

There were no *a priori* differences between the pro-saccade trials and the anti-saccade trials for the values or timings of the ten varied attributes. The only difference was that for the anti-saccade task the *Voluntary-Motor* and the *Inhibitory Gate* inputs were rotated 180° from what they were in the pro-saccade task because they were goal-driven inputs. In order to test the simplest form of the model, we initially did not add any task-based biases. Finally, at the start of each trial, Inputs 5, 7, and 8 were adjusted to their trial specific MaxVal settings. Both the ***c***^***int***^ and ***u***vectors were reset to their resting state of –30, as they represented more of a negative membrane potential as opposed to a firing rate. These steps were taken so that each trial was not affected by the preceding trial.

### Analysis of Human Behavior

To quantify whether the model's performance was adequate, we compared its performance to previously published saccadic behavior from a population of healthy human adults who performed pro- and anti-saccade tasks in a blocked design (Munoz et al., 1998). Saccadic behavior recorded from 73 human participants (aged 18–39) was divided into specific categories and each participant's data was normalized by the number of trials performed. For each participant, trials were split by task instruction (pro vs. anti). Anti-saccades were split by whether the saccade was executed to the correct or incorrect location (correct vs. error). Additionally, the correct pro-saccades and direction error anti-saccades were further subdivided by a temporal cut-off into two groups: “express” vs. “regular” latency saccades. Express latency saccades were defined as saccades initiated between 90 and 138 ms after stimulus appearance. Saccades initiated before 90 ms were not included in any analysis because they were equally likely to be made in either direction and were therefore classified as anticipatory (Munoz et al., 1998). The delineation between express and regular latency saccades was set to 138 ms, as it was a multiple of the bin size that was used (see below) and was closest to the previously used value of 140 ms (Fischer et al., 1993b; Munoz et al., 1998).

SRT histograms the five types of saccades were created using 6 ms bins. Normalized histograms for these saccades were created for each participant and then the average of those histograms was created to describe the population. The medians, means, and standard deviations of SRTs were calculated for comparison with data from the model. Additionally, the percentages for each behavior type were computed. Each value was calculated for each participant, and each participant's values were averaged to describe the population. To quantify the SRT variability difference between pro-saccades and anti-saccades, we created a variation difference score for each participant by subtracting the standard deviation of their Regular-Pro SRTs from the standard deviation of their Correct-Anti SRTs. This normalization process was done to control for inter-subject variations and isolate task differences. If the difference between these two standard deviations was negligible at the population level the outcome would create normally distributed values, centered on zero; a *t*-test was used to test this null hypothesis. Previously, measurements of average latency and variability were for Regular-Pro and Express-Pro combined. Here, they were separated because if they were grouped together, the measurements of the mean, median, and standard deviation for SRTs would have been affected by the percentage of Express-Pro, even though no change in the timing of Regular-Pro took place. By separating these saccades, we could show how SRT for regular latency saccades changed independently from the number of express latency saccades.

Finally, as the anti-saccade task is a competition between the automated drive to initiate a saccade toward a visual stimulus and a voluntary drive to initiate a saccade in the opposite direction, a subtraction of the cumulative curves for the correct anti-saccades and the direction-error saccades was done to estimate the point in time at which the voluntary signals started to overcome the automated signals. This anti-saccade difference curve was created from stimulus appearance (i.e., time zero) to 600 ms to measure ongoing performance. This is similar in concept to the Stop-Signal Reaction Time (Hanes and Schall, 1995, 1996; Stuphorn et al., 2000). Both are examples of measuring when a planned response can be canceled. As we have seen from human data (Munoz et al., 2007), anticipatory saccades were equally likely to be made in either direction, thus the difference curve remains near zero until express latency saccades begin to occur. As direction errors are generally earlier than correct anti-saccades, the difference curves should then dip below zero during the express latency window. Then, as correct anti-saccades start to outnumber direction errors, the difference curve should begin to rise again after the express window, eventually climbing above zero. The “Voluntary Override Time” was determined by first finding the global minimum turning point (i.e., the lowest value on the anti-saccade difference curve).Then finding the first subsequent point that increased by at least 1 from the value just prior to it. In other words, the velocity (1st derivative) of performance (correct-error) was equal or greater than 1.

### Analysis of Model Behavior

The model data was analyzed in the same manner as the human data so that the two could be compared. When the data was not normally distributed, nonparametric tests such as the Wilcoxon–Mann–Whitney-U test (WMWU) were used. In order to investigate what effects the variation of the 10 attributes (Table 1) had on SRT, all trials were sub-divided by the 3 settings for a given attribute (e.g., trials were sub-divided by the small, medium, or large values for the RoR of the *Automated Motor* input). This resulted in 10 regroupings of the full dataset; one for each manipulated attribute.

To quantify the effects of the controlled variability on SRT, the Kruskal-Wallis test was used to test for significant differences between the small, medium, and large sub-groups of each manipulated attribute. Due to the large number of trials that were collected, miniscule differences in median SRTs between groups would be construed as statistically significant. We therefore used the difference in median SRTs between the trials with small and large setting as a measure of absolute effect (Sullivan and Feinn, 2012). Cohen's d effect size calculations were also performed with different standard deviations for different behaviors.

To measure how variation of the 10 attributes determined which saccade the model selected, we measured the frequency of each setting for each saccade type. The values of the attributes were equally probable for each task, so if the attribute settings had no effect on saccade selection then all three values should be equally present for that type of saccade. If the proportions were not equal then it would reveal that the value of the attribute must have caused, or blocked, that behavior. To quantify the effects of the controlled variability on saccadic selection, the Chi-Square Goodness of Fit test was performed to test for significant difference from expected equivalence.

## Results

### The Model Reproduced the Five Types of Saccades Characteristic of Human Behavior

**Figure 5:**
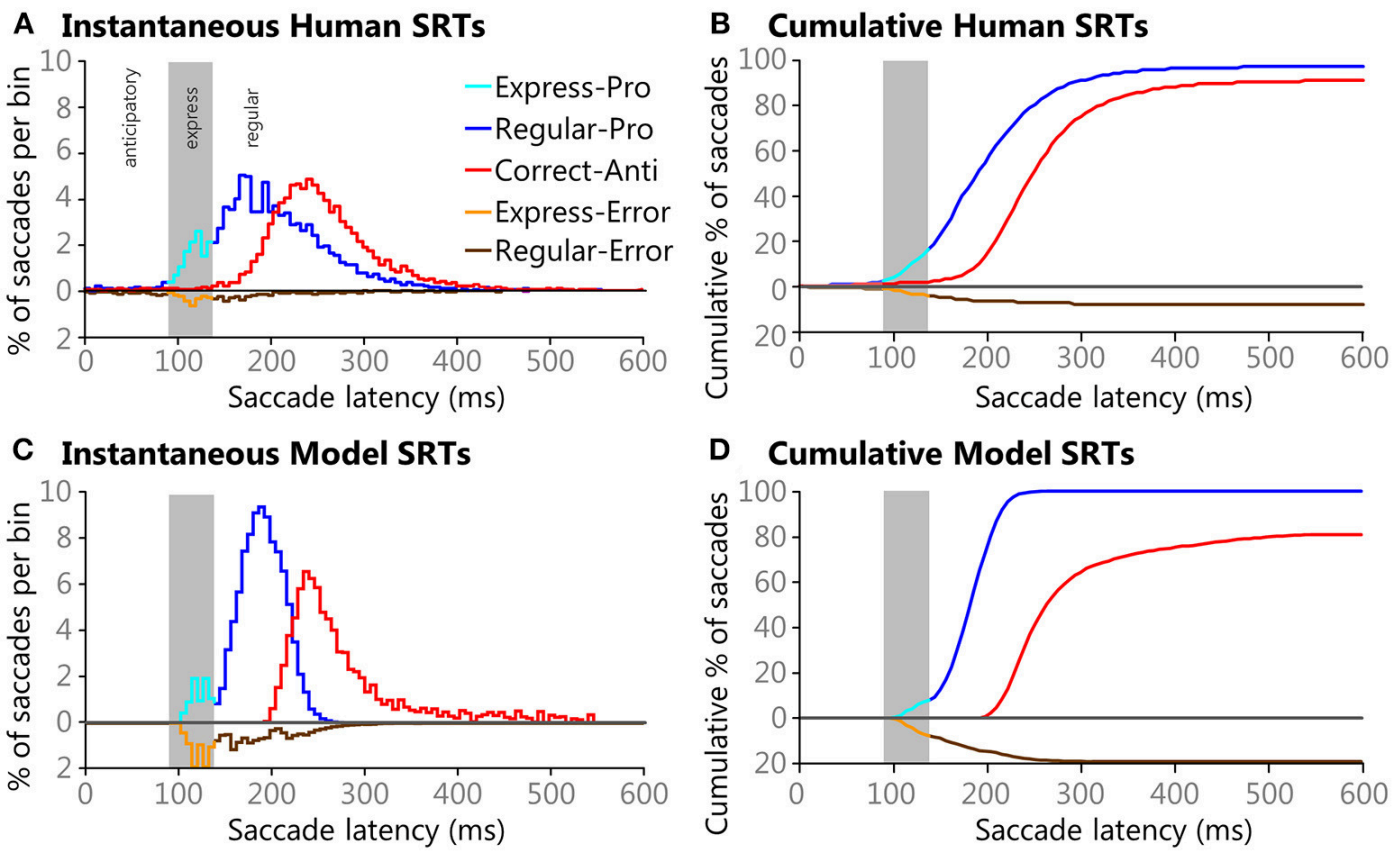
Histograms of Human **(A,B)** and Model **(C,D)** SRTs plotted using 6 ms bins. Direction errors are plotted below the zero line for ease of comparison. Gray areas in each pane indicate the express saccade window (90–138 ms). Instantaneous **(A)** and cumulative **(B)** histograms showing the percentages of saccades types and their latencies from healthy adults performing the pro- and anti-saccade tasks. Data is pooled from 74 participants between the ages of 18-39 that preformed a total of 14,493 saccades that fell into one of the five saccade types. Instantaneous **(C)** and cumulative **(D)** histograms showing percentages of saccades types and their latencies from the model. The model performed 118,098 trials, all of which had saccades that fell into one of the five saccade types.

Instantaneous and cumulative histograms showing the latencies of the five different types of saccades from the human data set (Munoz et al., 1998; Coe and Munoz, 2017) are presented in Figures 5A,B, respectively. The data were compiled from 74 participants, aged 18–39, who performed a total of 14,140 saccades that were classified into one of the five saccade types. Trial counts, percentages, medians, means, and standard deviations for SRT for the five types of trials are presented in Table 2 for the human data. Regular-Pro SRTs were earlier than Correct-Anti SRTs (*p* < 0.01 WMWU) and the variance analysis showed that Regular-Pro SRTs were less variable than Correct-Anti SRT (*p* < 0.001, *t*-test).

**Table 2:** Behavioral data from 74 participants aged 18–39 years.

		**Regular-pro**	**Express_pro**	**Correct-anti**	**Regular-error**	**Express_error**
**Total count**		4502	818	8171	390	259
Population means	Count	61.7	11.2	110.4	5.3	3.5
	Percent	86.6	13.4	92.3	4.7	3.0
	Median SRT	206.3	120.0	253.6	212.9	117.6
	Mean SRT	213.8	119.3	259.9	226.7	117.4
	STD SRT	42.0	10.7	50.1	63.6	10.0

The model reproduced the five types of saccades (Figures 5C,D) in a similar fashion, with reduced variability for the Regular-Pro SRT and higher anti-saccade error rates. Trial counts, percentages, medians, means, and standard deviations for SRT for the 5 types of trials are presented in Table 3 for the model data. Similar to the human data, the mean Regular-Pro SRT was earlier than the mean Correct-Anti SRT (*p* < 0.01 WMWU) and the Regular-Pro SRT was less variable than Correct-Anti SRT.

**Table 3:** Behavioral data from the model.

	**Regular-pro**	**Express_pro**	**Correct-anti**	**Regular-error**	**Express_error**
Count	54898	4151	47756	7142	4151
Percent	92.97	7.03	80.875	12.095	7.03
Median SRT	190	120	256	186	120
Mean SRT	190.36	122.20	276.98	194.82	122.20
STD SRT	22.45	8.28	66.38	38.75	8.28

### The Effects of Attribute Variability

**Figure 6:**
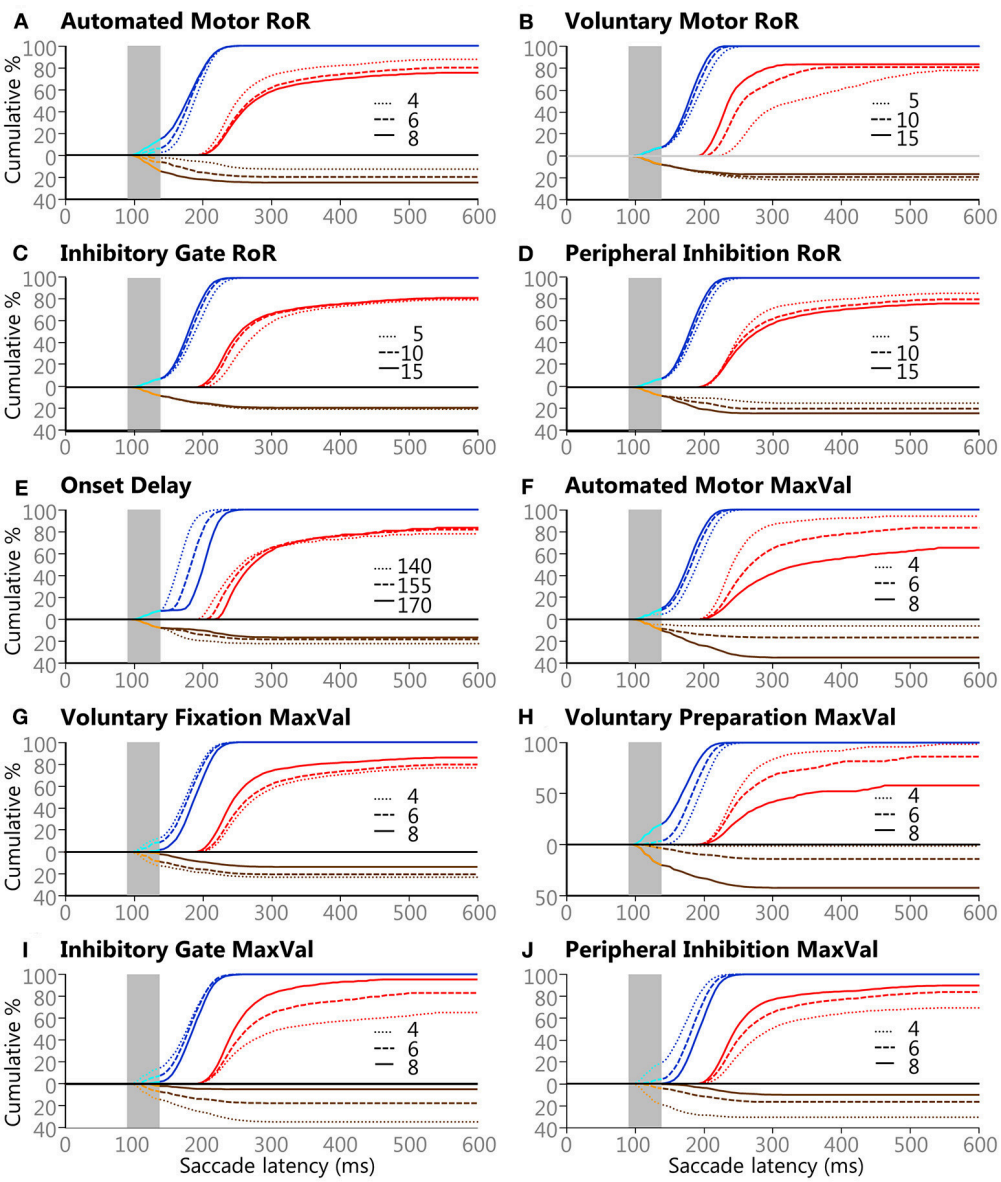
The effects of the 3 values for the 10 variables on saccade selection and latency. Panels **(A–J)** show the effects of the Controlled Deterministic Variability for each of the ten manipulated attributes described in the Methods section and Table 1. All blue lines are Regular-Pro, cyan lines are Express-Pro, red lines are Correct-Anti, brown lines are Regular-Errors, and orange lines are Express-Errors. All direction errors are plotted below the zero line for clarity. Solid lines indicate large values for each setting, dashed lines indicate medium value for each setting, and dotted lines indicate small value for each setting. Vertical gray areas indicate the express latency window.

A qualitative summary of the effects the manipulated attributes had on the model's behaviors is shown if Figure 6. This figure can also help to estimate what the outcome would have been if higher or lower values would have used (see Table 1). Each plot contains the same 118,098 trials as Figure 5D, but the data were sorted by the 3 settings for the variable in question. Some of the effects are straightforward. For example, increasing the Onset Delay for internally initiated inputs (Figure 6E) produced a clear rightward shift in SRT after the express window, indicating that a larger Onset Delay slowed SRT but had minimal effect on what type of saccade was selected, as measured by express latency saccade rate or error rate. On the other hand, increasing the MaxVal for the *Inhibitory Gate* input (Figure 6I) produced a clear vertical shift, during and after the express window, indicating that it had a strong determining force for what type of saccade was selected, as measured by the frequency of express latency saccades and direction errors. Furthermore, the MaxVal for the *Automated Motor* input (Figure 6F) influenced both SRT and saccade selection.

#### Saccadic Reaction Time Variability

Saccadic Reaction Time (SRT) variability was assessed for only the Regular-Pro, Correct-Anti, and Regular-Errors; as Express-Pro and Express-Errors were confined within a fixed time-window. The results from the Kruskal-Wallis test on SRT variability showed that all but two median SRT comparisons were significantly affected by the variability of the attributes (*p* < 0.001). However, due to the massive size of the data set, any absolute difference greater than 3 ms constituted statistical significance. Thus, the difference in median SRT between small and large values (i.e., the median-shift) was used as a measure of absolute effect (Table 4). Cohen's *d* effect size analysis (Table 5) uses standard deviation to normalize the effect size results. This creates two caveats for the analysis of our data, the second one being the most important. First, each behavior had very different standard deviations giving each behavior different effect size scores for the same effect. Second, the data is so skewed that the standard deviation is not a great descriptor of the data. This is especially the case for Correct-Anti histogram as it has a much larger standard deviation due to the very elongated tail. Skewness for Regular-Pro, Correct-Anti, and Regular-Errors are: 0.15, 1.85, 0.61 respectively, where over 1 is considered very skewed and 0.5 to 1 is moderately skewed. These descriptive statistics were designed for a more normal distribution so here the exaggerated standard deviation minimizes Cohen's d effect size especially for Correct-Anti. Using a single standard deviation for the three behaviors combined would be similar to using absolute median-shift, except that the effect values would lose their intrinsic meaning. Although both results are presented the absolute effect of the median-shift (in milliseconds) was used to discuss how each setting influenced SRT.

**Table 4:** Median SRT shift results (Δms).

**Attributes**	**Regular-pro**	**Correct-anti**	**Regular-error**
Onset delay	37^§^	21^§^	50^§^
Auto-motor RoR	−7	11^*^	−55^§^
Vol-motor RoR	−11^*^	−56^§^	−16^§^
Inhibit-gate RoR	−9^*^	−21^§^	−3
Periph-inhib RoR	−8	7	−54^§^
Auto-motor MAX	−13^*^	32^§^	28^§^
Vol-fixation MAX	9	−18^*^	−3
Vol-prep MAX	−20^§^	14^*^	22^§^
Inhibit-gate MAX	5	−11^*^	−19^§^
Periph-inhib MAX	17^*^	−20^§^	53^§^

* indicates moderate effect (30–60th percentile)

§ indicates major effect (60–100th percentile)

*The difference in median SRT between the large setting and the small setting was used as a measurement of absolute effect size (Sullivan and Feinn, 2012). Any absolute value greater than 3 ms was significant (p < 0.01)*.

**Table 5:** Cohen's d effect size calculations for each behavior $\frac{large-small}{\sigma }$.

**Attributes**	**Regular-pro (σ = 22.45)**	**Correct-anti (σ = 68.38)**	**Regular-error (σ = 38.75)**
Onset delay	1.65	0.32	1.29
Auto-motor RoR	−0.31	0.17	−1.42
Vol-motor RoR	−0.49	−0.84	−0.41
Inhibit-gate RoR	−0.40	−0.32	−0.08
Periph-inhib RoR	−0.36	0.11	−1.39
Auto-motor MAX	−0.58	0.48	0.71
Vol-fixation MAX	0.40	−0.27	−0.08
Vol-prep MAX	−0.89	0.21	0.57
Inhibit-gate MAX	0.22	−0.17	−0.49
Periph-inhib MAX	0.76	−0.30	1.37

Negative scores indicate that the large setting reduced SRT relative to the small setting (i.e., bigger was faster). As the absolute effect (in milliseconds) is constrained by the variability of the input settings, we delineated the results by the 30th and 60th percentile rank of the absolute median-shift. This corresponded to a difference less than 10 ms as a minor effect and a difference above 20 ms as a major effect. Only moderate (from 10 to 20 ms) and major effects are discussed.

The Regular-Pro SRTs were moderately affected by 3 attributes, and 2 attributes had major effects (Table 4). Not surprisingly, largest effect on Regular-Pro SRT was due to the Onset Delay; the later the inputs started, the slower the model was to select a saccade. This was true for Regular-Pro, Correct-Anti, and Regular-Errors. Also unsurprising, was the effect of the *Voluntary Preparation* MaxVal, and *Automated Motor* MaxVal, which reduced Regular-Pro SRT when they were stronger. This confirms that stimulus predictability (as modeled by the *Voluntary Preparation* input) and training (as modeled by the *Automated Motor* input) reduced SRT. Additionally, an increase in the *Voluntary-Motor* RoR moderately decreased Regular-Pro SRT. Lastly, an elevated *Peripheral Inhibition* MaxVal had a moderate slowing effect on Regular-Pro SRT.

The Correct-Anti SRTs were moderately affected by 4 settings, and 5 settings had major effects (Table 4). A larger *Voluntary-Motor* RoR and the *Inhibitory Gate* RoR decreased the model's Correct-Anti SRTs. There were also faster SRTs when the *Voluntary Fixation* MaxVal, the *Inhibitory-Gate MaxVal*, and *Peripheral Inhibition* MaxVal were larger. Lastly, a larger Onset Delay, *Automated Motor* MaxVal had a major slowing effect on SRT, whereas Auto-Motor RoR and Voluntary *Preparation* MaxVal had only a moderate slowing effect on Correct-Anti SRTs.

Regular-Errors SRTs were moderately affected by one setting, and 8 settings had major effects (Table 4). Regular-Errors occurred later (i.e., a longer competitive process between the automated and voluntary inputs) when the Onset Delay, *Automated Motor* MaxVal, the *Voluntary Preparation* MaxVal, and the *Peripheral Inhibition* MaxVal were larger. However, Regular-Errors occurred earlier when the *Automated Motor* RoR, the *Voluntary Motor* RoR, the *Peripheral Inhibition* RoR, and the *Inhibitory Gate* MaxVal were larger. One rather interesting aspect about the difference between the model and the human Regular-Errors SRT distributions is evident in Figures 5A,C. Just after the 200 ms time point, the histogram for the human data approached zero for Regular-Errors SRTs but the histogram for the model shows that another group of direction error saccades were made. A *post-hoc* investigation addresses this discrepancy below.

#### The Origins of Saccadic Action Selection

**Figure 7:**
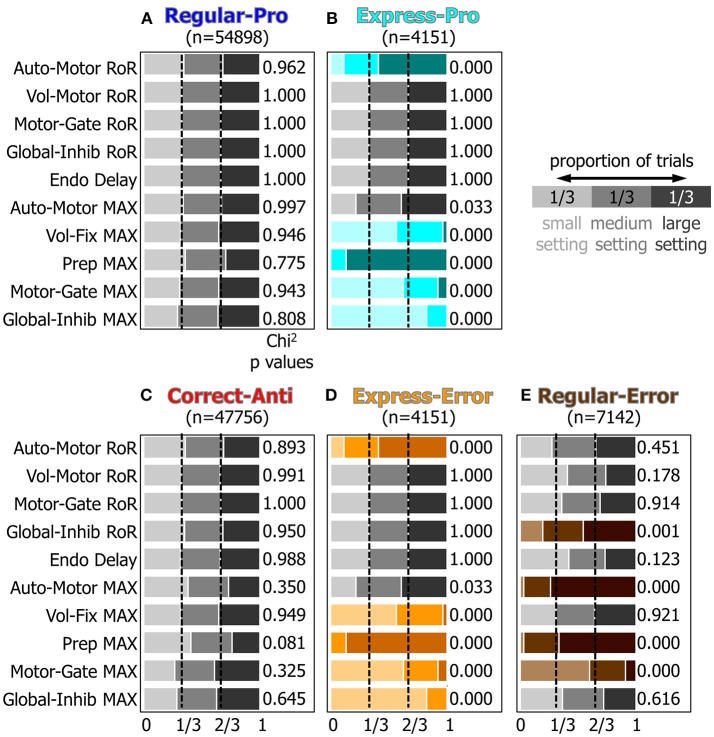
A visualization of the results of the Chi^2^ test for the 10 manipulated attributes. Panels **(A–E)** represent how the ten manipulated attributes, described in the Methods section and Table 1, influenced each behavior's probability. Within each behavior, the proportion of small, medium, and large settings should be equal if that input's setting had no effect on saccade selection. Colored rows indicate which settings had results that were significantly different from the expected proportions of 1/3 for each setting (*p* < 0.001, chi^2^ test).

To measure how the small, medium, and large settings for the attributes affected which type of saccade was selected a Chi-Squared analysis was performed (see methods). Figure 7 graphically represents the results from the Chi-Squared analysis and rows with color indicate which attributes had a significant (*p* < 0.001) effect on action-selection.

For Regular-Pro trials (Figure 7A) all attributes had equal distributions of small, medium, and large values. This established a baseline and indicated that no single setting was responsible for selecting Regular-Pro.

For Express-Pro trials (Figure 7B) there were significant shifts in several of the attribute settings. When the model made an Express-Pro, the *Automated Motor* RoR and the *Voluntary Preparation* MaxVal were predominantly large values. Whereas the MaxVal for the *Voluntary Fixation, Inhibitory Gate*, and *Peripheral Inhibition* inputs were predominantly small values. In fact, when the *Voluntary Preparation* MaxVal was at its lowest setting or the *Peripheral Inhibition* MaxVal was at its highest setting, this model did not make any Express-Pro. This is in agreement with the findings that express latency saccades are facilitated by stimuli that appear in predictable and practiced locations (Paré and Munoz, 1996; Sparks et al., 2000) and are associated with a the buildup of preparatory activity (Dorris and Munoz, 1998). For Express-Errors (Figure 7D), the shifts in the settings were identical to the Express-Pro results.

For Correct-Anti trials (Figure 7C) the proportions of attribute settings were not significantly different from the null hypothesis, although one minor tendency was present. In other words, the number of correct anti-saccades did not depend on only one of the inputs. The *Voluntary Preparation* MaxVal was more frequently a small setting (*p* = 0.081, Chi^2^), showing that the model was more likely to make a successful anti-saccade if the anticipatory build up activity for a visual stimulus (i.e., the *Voluntary Preparation* input) was reduced.

Regular-Errors trials (Figure 7E) also showed significant shifts in several of the attribute settings. The *Automated Motor* MaxVal and the *Voluntary Preparation* MaxVal were predominantly large settings. This shows that the model made more Regular-Errors when the automated drive to make a saccade to a visual stimulus was augmented by increased anticipatory build up activity. Additionally, the MaxVal attribute for the *Inhibitory Gate* input was predominantly small, showing that if the inhibition, which needed to be removed to unmask a motor command, was weak, then the model was more likely to make Regular-Errors.

### *Post-hoc* Investigations

The primary simulation was run with the basic assumptions of the model and without any biases between pro-saccade trials and anti-saccade trials. However, the results from the primary simulation lead to several *post-hoc* simulations and improved behavioral performance, i.e., better matched human behavior. Three *post-hoc* investigations (with several simulations each) were performed to test findings and improve the model's performance. The first investigation addressed the source of the temporal difference between the pro and anti SRTs. The second addressed the difference between the frequency of express latency saccades between the model's performance and the human behavior. The final investigation addressed the greater number of longer latency (>200 ms) direction errors exhibited by the model in comparison to the human data.

#### Post-hoc Investigation 1

In this model and others (Cutsuridis et al., 2007; Cutsuridis, 2017), the delay of the anti-saccade SRTs was not caused by a predetermined delay imposed specifically on anti-saccade trials. Here, the temporal delay in anti-saccade SRT was caused by a conflict between voluntary and automated processes. To validate this finding, we ran additional simulations with modified settings. In the first *post-hoc* simulation, the *Automated Motor* input was removed by setting its RoR = 0 (it never rose above zero). This caused the model to perform both tasks perfectly (i.e., zero direction errors) and drastically reduced the difference between the Regular-Pro and the Correct-Anti SRT distributions (mean Regular-Pro SRT: 220 ms, mean Correct-Anti SRT: 227 ms). In this first *post-hoc* simulation, some Correct-Pros were earlier due to some residual activity from the Visual Transient. This does not mean that the Visual Transient caused the saccades, merely that the residual effects of the visual stimulation raised the Neural Field to a higher state of excitability at the location of the stimulus. Thus, the subsequent motor commands toward the stimulus started at a slightly higher level, which pushed them across the threshold earlier. However, subsequent motor commands on the opposite side of the stimulus started at a slightly lower level of excitability, which forced them to cross the threshold later. The second version of this simulation removed both the *Automated Motor* and the Visual Transient inputs by setting their RoR = 0. With both of these inputs reduced to zero, the Regular-Pro and the Correct-Anti SRT distributions were identical to the millisecond (mean SRT: 225 ms), as they both were solely driven by voluntary motor commands and the model contained no noise.

#### Post-hoc Investigation 2

In the human data there were more Express-Pro than Express-Errors (Figure 5A). For our model, however, the number of Express-Errors was identical to that of the Express-Pro (Figure 5C) because the settings that caused these behaviors were equally present in both tasks by design. In a trained participant, however, there has been shown to be a difference in the “intentional state” between pro and anti-saccade tasks in the FEF, SEF, and IntraParietal Sulcus (IPS) of humans (Curtis and D'Esposito, 2003; Ford et al., 2005) and in single unit recordings in both FEF and SC (Everling et al., 1999; Munoz et al., 2000). Everling et al. (1999) showed that anti-saccade trials had enhanced activity over pro-saccade trials in fixation neurons whereas anti-saccade trials had less activity than pro-saccade trials in saccade neurons.

In an effort to recapitulate an “intentional state” task-dependent strategy (or bias) in our dataset, we removed trials with specific settings for fixation and inhibitory inputs. The removal of pro-saccade trials with large MaxVal settings for the *Voluntary Fixation, Inhibitory Gate*, and *Peripheral Inhibition* inputs emulated a reduction in average fixation and inhibition activity during pro-saccade trials. We also removed the anti-saccade trials with the small MaxVal settings for the same three inputs to emulate an increase in average fixation and inhibition activity during anti-saccade trials. This dramatically changed the percentages of express latency saccades from being equal at 7.03% for both Express-Pro and Express-Errors and 12.10% Regular-Errors with full settings, to 20.95% for Express-Pro, 0% for Express-Errors, and 6.05% Regular-Errors. In fact, removing the pro-saccade trials with the large MaxVal setting and the anti-saccade trials with the small MaxVal setting for any one of these inputs resulted in there being more than twice as many Express-Pro than Express-Errors. The *Voluntary Fixation* MaxVal manipulation resulted in 10.13% Express-Pro vs. 4.58% Express-Errors, and 12.52% Regular-Errors. The *Inhibitory Gate* MaxVal manipulation resulted in 9.72% Express-Pro vs. 3.96% Express-Errors, and 7.26% Regular-Errors. The *Peripheral Inhibition* MaxVal manipulation resulted in 10.54% Express-Pro vs. 1.85% Express-Errors, and 11.53% Regular-Errors. These modified results better matched the human results, with 17.6% Express-Pro vs. 2.9% Express-Errors and 4.3% Regular-Errors. Although the manipulation of each of these three inputs improved the model's performance, it was the *Voluntary Fixation* manipulation that was most specific to express latency saccades as it had the least effect on the percentage of Regular-Errors and the Regular-Errors SRT. For comparison, the standard model's Regular-Errors SRT was 195 ms (STD = 39 ms), and the *Voluntary Fixation* manipulation's mean Regular-Errors SRT was 196 ms (STD = 39 ms), but the *Peripheral Inhibition* manipulation's mean Regular-Errors SRT was 204 ms (STD = 39 ms).

#### Post-hoc Investigation 3

**Figure 8:**
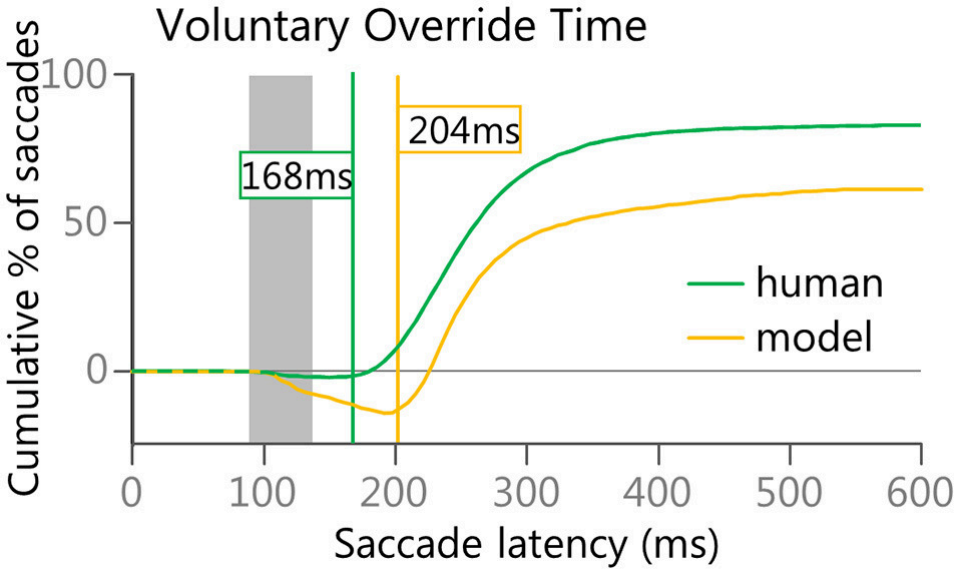
Voluntary Override Time was calculated to estimate when voluntary signals started to override automated signals. The lines in Figure 8 are subtractions of the Red and Orange/Brown lines in Figures 5B,D.

The “Voluntary Override Time” analysis (Figure 8) indicated that the model took longer than the human participants to get the voluntary signals to override the automated signals. The human participants averaged only a 168 ms delay where as the model averaged 204 ms delay. This was due to the greater number of longer latency (>200 ms) direction errors in the anti-saccade task for the model (Figure 5C) than for the human population (Figure 5A). As discussed earlier, regular latency saccades have been described as “fast-regular” saccades and “slow-regular” saccades. We hypothesized that the “early” direction errors could be due to a lack of general inhibition, whereas the “late” direction errors could be due to a lack of specific inhibition of the automated signal. For the model, the *Automated Motor* input was fashioned after signals with an initial response that was not informative as to whether or not a saccade was going to be made to a specific stimulus, and we initially emulated the simplest form of this concept. However, in most situations the firing patterns of neurons carrying this early component alter their firing patterns to reflect intended motor plans (Snyder et al., 1997; Gold and Shadlen, 2007; Andersen and Cui, 2009; Bisley and Goldberg, 2010) and even perceived value (Sugrue et al., 2004). This response inhibition and modulation is fundamental in performing complex tasks (Funahashi, 2001; Alahyane et al., 2014) and we posit that voluntary signals help reshape the automated response.

Our final *post-hoc* investigation included something similar to “crosstalk” between the *Voluntary Motor* input and the *Automated Motor* input prior to the summation of all inputs. At each iteration (or time point), a response inhibition instruction was created by horizontally flipping the array that represented the *Voluntary Motor* input and subtracting 25% of those values from the *Automated Motor* input. Any values below zero in the adjusted *Automated Motor* input were set to zero, as this input was not designed to be inhibitory. This response inhibition used an inverted *Voluntary Motor* input because it would commence with Onset Delay, as all other voluntary inputs did, and its strength would vary with the strength of the *Voluntary Motor* input. This had the effect of reducing the strength of the *Automated Motor* input only after the Onset Delay but had no effect on the *Voluntary Motor* input. This method was chosen because it was simple and followed the assumption that any such response inhibition would be driven by dynamic voluntary signals, both in timing and in strength, and theoretically could be implemented using a process similar to inhibitory interneurons. This inhibitory crosstalk had the effect of reducing the Regular-Errors specifically. Most importantly, this effect was more pronounced for the Regular-Errors after 200 ms and resulted in behavior that better approximated the human data. To quantify this, the ratio between early (140–199 ms SRT) and late (200–259 ms SRT) Regular-Errors was calculated using the count of all saccades in the two time windows. For the human data there was a 3:1 ratio of “early” to “late” Regular-Errors. In the original simulation there was a 1.8:1 ratio of “early” to “late” Regular-Errors whereas with the inhibitory crosstalk simulation there was a 3.1:1 ratio of “early” to “late” Regular-Errors.

**Figure 9:**
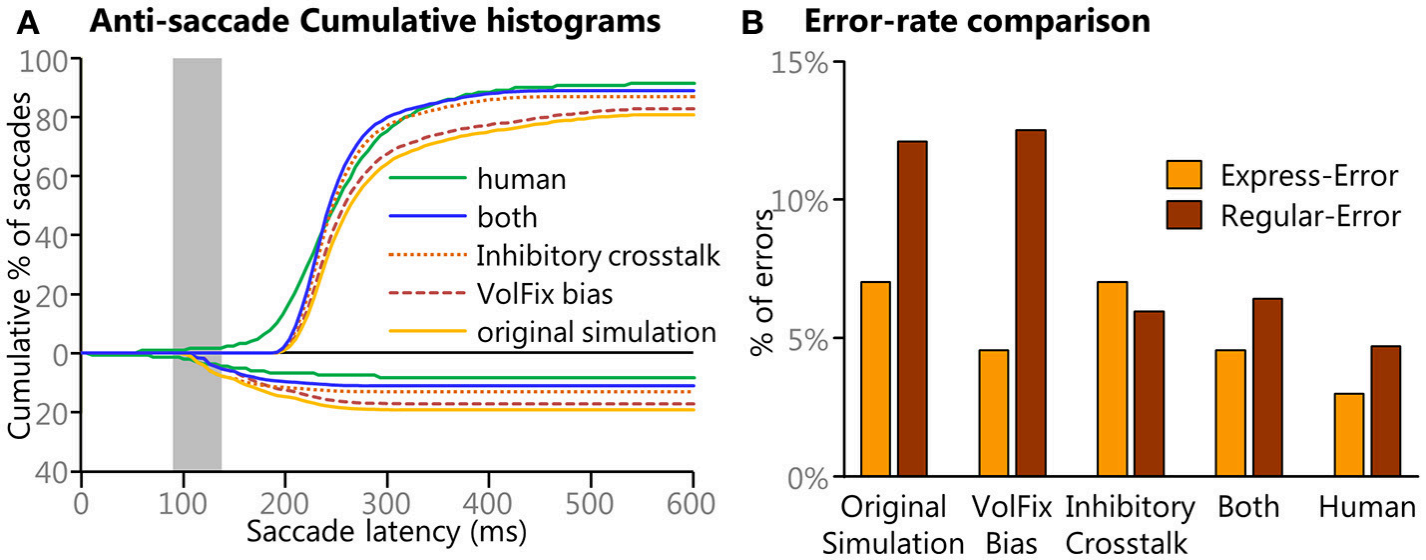
Results from the different simulations of the model. **(A)** Cumulative histograms of SRTs for the results from the original settings and for the *post-hoc* manipulations are compared to the human data. A clear progression of improvement is noticeable. **(B)** To better clarify the specific results from the manipulations bar graphs of the different direction errors are presented. Here we show how modulation of the levels of fixation activity modulated the frequency of express latency saccades and how the results from the “cross-talk” modulation that emulated a cancel contribution specifically targeted the later (>200 ms) direction errors. The simulation with both modulations better emulates the human data.

Figure 9 shows how the final two *post-hoc* investigations improved the model's performance to better emulate the human data. Figure 9A shows cumulative histograms of the model's performance with original, the individual manipulations, the combined manipulations, and the human data. Only anti-saccades are shown as there was only a miniscule change to the pro-saccades. Figure 9B quantifies the error rates and shows how the simulation that used both modifications better resembled the human behavioral results.

## Discussion

Our model helps to shed insight into how the spatial and temporal aspects of multiple afferent signals, from numerous brain areas, can coalesce in the SCi and interact to guide saccadic action selection and response inhibition. The model not only accrued information for complex decision-making processes, but also reacted quickly in response to sensory information to select automated, or even reflexive, saccades. The model replicated the well-known effect of correct anti-saccades having slower SRTs than correct pro-saccades (Fischer and Weber, 1992; Munoz et al., 1998; Peltsch et al., 2011). This difference was an emergent property of the internal competition, vs. collaboration, between the automated and voluntary commands, which either slowed or quickened SRT, respectively. With the introduction of multiple “signal-inspired” inputs, our model outperformed the previous version (2001) in its ability to match human behaviors, specifically in producing express and regular latency direction errors in the anti-saccade task as well as express and regular latency pro-saccades. This strategy of using numerous inputs is less parsimonious, but the improved performance and the added ability to modulate inputs individually, testing specific hypotheses, we believe justify the added complexity. More complicated models exist and some can replicate express and regular latency pro-saccades (Heinzle et al., 2007; Wiecki and Frank, 2013; Lo and Wang, 2016) but to the best of our knowledge, the only other model to simulate more robust behavioral diversity is an intricate model of spiking neural circuits (Lo and Wang, 2016). This intricate decision-making model simulated express and regular latency for correct pro-saccades and direction errors as well as modulating behavior by changing tasks and input strength parameters.

The relatively simple nature of our inputs and controlled variably of the attribute settings enabled us to trace backwards from a particular behavior to understand what caused each behavior. This in turn led to *post-hoc* simulations that further improved performance via the modulation of the inputs and were based on neurophysiologically inspired hypotheses. Preliminary *post-hoc* investigations provided a rationale for why anti-saccades were slowed and why pro-saccades were sped up, and the sources of express latency pro-saccades and direction errors (both express and regular latency). Further simulations showed how a pre-emptive bias affected express saccade prevalence for each task, and how an inhibitory crosstalk between inputs not only improved direction error rates, but also improved the similarity between human and model behaviors.

There are still numerous ways to further test and improve the model and its inputs that are too expansive for this introductory setting. One behavior that was not emulated was a corrective anti-saccade after a direction error, which has been modeled before (Cutsuridis et al., 2007). In the current rendition, the *Voluntary Motor* input was quenched once a saccade was made but this could be altered to be dependent on the outcome of the saccade.

### What Drives Express Latency Saccades?

The model's results showed that express latency saccades involve the same mechanisms in both tasks, but it was the difference in “preparatory bias” between tasks that affected their frequency, as has been seen in human fMRI (Connolly et al., 2002). The model recreated the well-known bi-modality of express latency and regular latency saccades in the pro-SRT distribution (Fischer and Weber, 1993), and is also in agreement with previous work, which showed that express latency saccades are produced via separate mechanisms than the regular latency saccades in both single unit recordings (Fischer and Weber, 1992; Paré and Munoz, 1996) and modeling (Cutsuridis et al., 2007). Although the original simulation of the model failed to emulate the imbalance in Express-Pro and Express-Errors seen in the human data (Figures 5A,C), *post-hoc* simulations did. We emulated task-dependent strategies to imitate an “intentional state” bias between pro- and anti-saccade tasks (Everling and Munoz, 2000; Curtis and D'Esposito, 2003) by removing certain settings for fixation and inhibition inputs. This bias recapitulated Express-Pro being more prevalent than Express-Errors similar to the human population data. In other words, the mechanisms behind Express-Pro and Express-Errors are the same, but it is the “intentional state” bias that determines their difference in frequency. The manipulation to induce an “intentional state” bias also influenced Correct-Anti SRT. This effect can also be seen in Figures 6I,J, solid lines; the more inhibition, the smaller the difference between pro-SRT and anti-SRT. Similar findings have been published in human studies (Olk and Kingstone, 2003). The model also showed that this “intentional set” bias can be produced by modulating either fixation or inhibition inputs. However, fixation inputs affected the express saccade rates with less effect on overall direction error rates. It is well known in the literature that a blank period between the disappearance of the fixation point and the peripheral stimulus is the best way to elicit express latency saccades [i.e., the gap effect, (Saslow, 1967; Fischer and Boch, 1983; Fischer and Ramsperger, 1984), which further supports that adjusting the fixation input was the most appropriate way to modify express latency saccade rate. This model explains how “reflexive” express latency saccades are the culmination of: preparatory build-up activity, reduced inhibition, lowered fixation activity, and of course a sensory response. All of which must coalesce in the SCi; without which reflexive express saccades cannot be made (Schiller et al., 1987).

### The Anti-saccade Task: Competition Between Automated And Voluntary Saccade Plans

In the anti-saccade task, the model's *Voluntary Motor* and *Automated Motor* inputs vied for saccadic control between two mutually exclusive actions. A stronger *Automated Motor* not only increased Correct-Anti SRT but caused Regular-Errors when the *Automated Motor* command won.

Another way to compare anti-saccade performance for human and model data was the “Voluntary Override Time” analysis. The anti-saccade difference curves (Figure 8) illustrated why our model had a higher direction error rate, specifically Regular-Errors. The effects of the *Automated Motor* input persisted too long as demonstrated by the delayed recovery of the model curve vs. the human curve. An actual “cancel” signal may not be necessary to perform the anti-saccade task, as shown in the original simulation, but it appears necessary in order to improve performance, especially for Regular-Errors after 200 ms post-stimulus. Such a cancel signal may come in the form of a reshaping of automated signals via voluntary signals in frontal and parietal cortices, or it might be a spatially focused inhibitory signal sent to the SCi from the basal ganglia (Watanabe and Munoz, 2011; Amita et al., 2018; Hikosaka et al., 2018b). A focused inhibitory signal sent to the SCi from the basal ganglia would more likely occur in a situation like the Stop-Signal saccade task, when there is a need to cancel a voluntary motor plan. For the anti-saccade task, we hypothesize that the voluntary signals of the frontal-parietal network are crucial for quenching the automated signals. Thus, we implemented a “crosstalk” style inhibition from the *Voluntary Motor* input on the *Automated Motor* input just prior to input summation and entry to the main model. This had a very specific effect of reducing later (>200 ms) direction errors in the anti-saccade task and further improving the model's performance.

Some remaining differences between the human data and the model data are in the variability and the delayed voluntary inputs. This made the model incapable of simulating Correct-Anti prior to 200 ms; evident in the human data. For these simulations there was a desire to investigate the express latency saccades independent of very fast automated or even voluntary saccades, thus we kept the voluntary inputs delayed. Another issue is the difference in variability in Regular-Pro SRT. Again, the desire to investigate the express latency saccades independently barred us from introducing a greater number of settings for the Onset-Delay, voluntary, and automated inputs.

## Conclusion

This platform of using separate “component-based” inputs has numerous possibilities for future work. Here, the age range of the non-clinical human data was from 18 to 39 years but future work could involve modulating the inputs to emulate child development (by modulating inhibition and fixation inputs), aging (by modulating accuracy of voluntary inputs), and neural degeneration (by modulating dis-inhibition regulation), to name a few. In fact, the inspiration to create this model was to set it up for testing a large range of hypotheses about the formation and degeneration of neural pathways and circuits.

## Author Contributions

BC was the main author, programmer, and human behavior specialist. TT was the original creator of the model and was instrumental in verifying updated equations and algorithms used. DM was the neurophysiology specialist.

### Conflict of Interest Statement

The authors declare that the research was conducted in the absence of any commercial or financial relationships that could be construed as a potential conflict of interest.
